# Spontaneous Rupture of the Urinary Bladder in an Elderly Diabetic Male

**DOI:** 10.7759/cureus.46481

**Published:** 2023-10-04

**Authors:** Adnan A Al-Nahawi, Abdulrhman M Alsuwailim, Ali S Alhassawi

**Affiliations:** 1 Urology, Almoosa Specialist Hospital, Al Ahsa, SAU

**Keywords:** abdominal wall swelling, ruptured bladder, urinary ascites, spontaneous bladder rupture, srub

## Abstract

Spontaneous rupture of the urinary bladder (SRUB) represents an infrequent but critical urological crisis with significant morbidity and mortality risk especially in cases of septicemia. While various factors contribute to its etiology, SRUB often manifests secondary to pre-existing bladder pathologies such as chronic inflammation, neoplasia, iatrogenic radiation exposure, or obstructive uropathy. An 82-year-old male presented with acute, left-lateralized abdominal discomfort. Clinical evaluation revealed diffuse erythema and swelling within the left lower abdominal quadrant, indicative of cellulitis. Pelvic sonographic imaging detected a 4 cm fluid collection, coupled with cellulitis in the left anterolateral segment of the lower abdominal wall, stemming from a discernible defect in the anterosuperior aspect of the bladder. Drainage of 1600 cc of purulent urine was achieved via a 16-Fr urethral catheter (Safety Science Medical Company, Riyadh, Saudi Arabia). Subsequent pelvic computed tomography and cystographic studies elucidated a pathological communication between the anterior bladder wall and the left lateral abdominal wall, along with a localized urinoma. The present case underscores the imperative nature of immediate therapeutic intervention in the effective management of SRUB. Successful surgical repair and a complication-free postoperative trajectory were observed, enriching the prevailing medical literature on SRUB. The case amplifies the necessity for acute awareness, expedient diagnostic procedures, and urgent surgical intervention as key elements in optimizing patient outcomes.

## Introduction

Spontaneous rupture of the urinary bladder (SRUB) is an uncommon and perilous medical condition characterized by a tear in the bladder wall without any external trauma. It is a rare and life-threatening condition, making up less than 1% of all bladder injuries [1]. Common presentations of SRUB include abdominal discomfort, emesis, and urinary retention. The lack of specificity in these clinical signs frequently complicates the diagnostic process, as they may mimic various other medical ailments. In general, individuals experiencing a rupture typically exhibit symptoms such as a sudden-onset of abdominal pain, impaired kidney function, and the presence of systemic infection [2].

The definitive diagnosis of SRUB is made by diagnostic imaging, such as an abdominal computed tomography (CT) scan or urinary cystograms [3]. Unfortunately, the diagnosis and treatment of SRUB are often delayed and missed; most cases are discovered during laparotomy. Surgical management is the recommended treatment for SRUB [3,4]. For the surgical approach, the type of surgical repair depends on the location and extent of the rupture [5]. The overall prognosis for SRUB is generally favorable, with a high likelihood of complete recovery for the majority of patients. Nonetheless, the risk of mortality substantially increases in instances where sepsis is present [1,4].

The significance of this case report is that it highlights the importance of considering SRUB in the differential diagnosis of patients with abdominal pain. This case also highlights the importance of early surgical intervention in patients with SRUB, as this can improve the chances of a good outcome.

## Case presentation

An 82-year-old male patient with a medical history of type 2 diabetes mellitus, hypertension, and chronic anemia attributed to small bowel arteriovenous malformation and benign prostatic hyperplasia (60 cc in size) presented with an acute episode of abdominal pain. For his past surgical history, he underwent a right hemiarthroplasty due to a fractured right hip three years ago. Previously, he experienced a one-time episode of urine retention more than one year ago when he visited the emergency department and was treated by inserting a silicone Foley catheter (Safety Science Medical Company, Riyadh, Saudi Arabia) for one week without identifiable cause as he did not follow-up with the urology clinic appointment. Upon arrival at the emergency department, he complained of severe left-sided abdominal pain. Physical examination revealed that the patient was conscious, alert, and oriented but appeared ill. The left lower quadrant showed tenderness, significant swelling, and poorly defined margins of erythema. The laboratory results were as follows: hemoglobin (Hb): 10.2 g/dL, white blood cells (WBC): 9.98 x 10^3^/uL, platelets (PLT): 218 k/uL, segmented neutrophils: 90%, and C-reactive protein (CRP): 270 mg/L.

Abdominopelvic ultrasound revealed bilateral mild to moderate hydronephrosis, distention of the urinary bladder (UB) with 1057 cc of turbid fluid containing precipitated debris, a 4 cm defect in the anterosuperior aspect of the bladder, and consequent fluid collection and cellulitis in the left anterolateral aspect of the lower abdominal wall. A 16 Fr urethral catheter was inserted and drained approximately 1600 cc of turbid urine (consistent with pure pus). His first urine culture was taken on the first day at the emergency department. The culture was identified as extended-spectrum beta-lactamases (ESBLs)-producing *Klebsiella pneumoniae*, leading to the initiation of intravenous ertapenem (1 gm daily).

**Figure 1:**
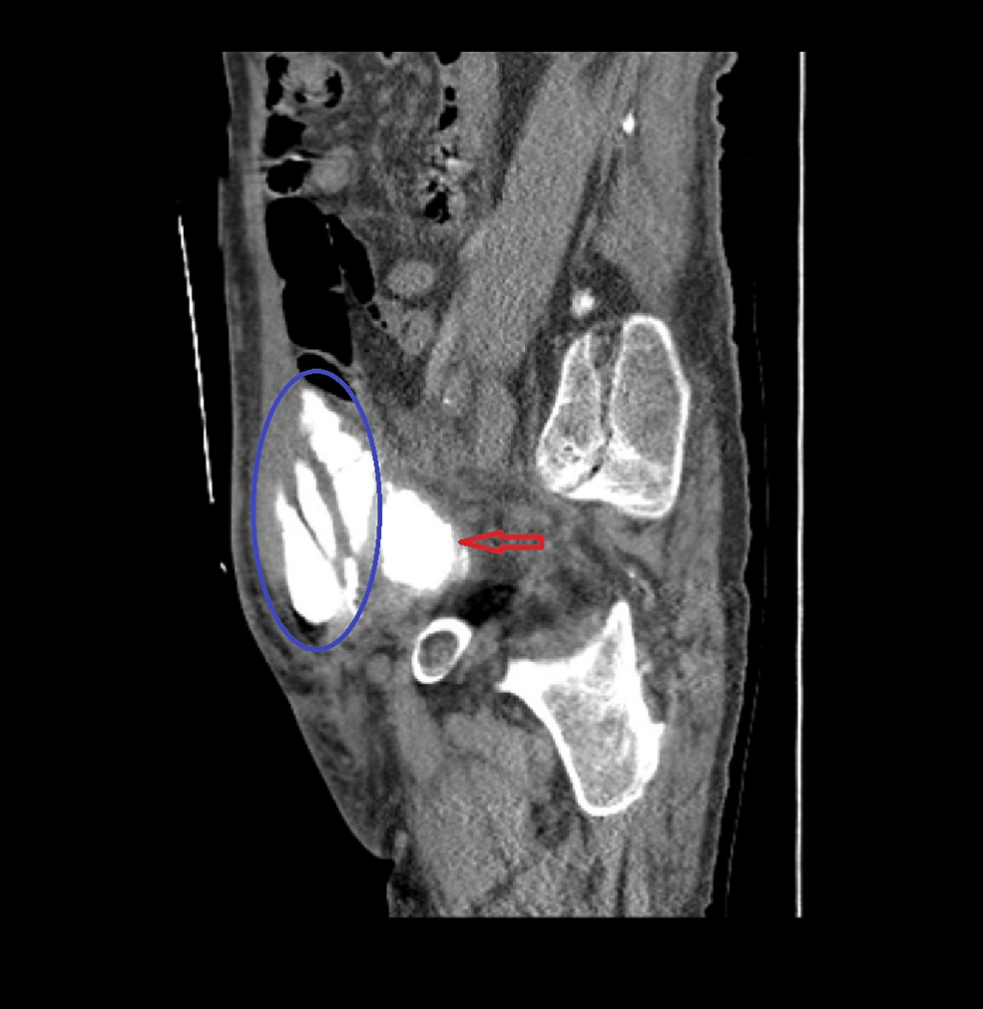
Computed tomography (sagittal view) study shows urinary bladder anterior superior wall large defect (red arrow) measuring about 4.3 × 4 cm communicating with anterior pelvic encysted fluid complicated localized urinoma (blue circle).

**Figure 2:**
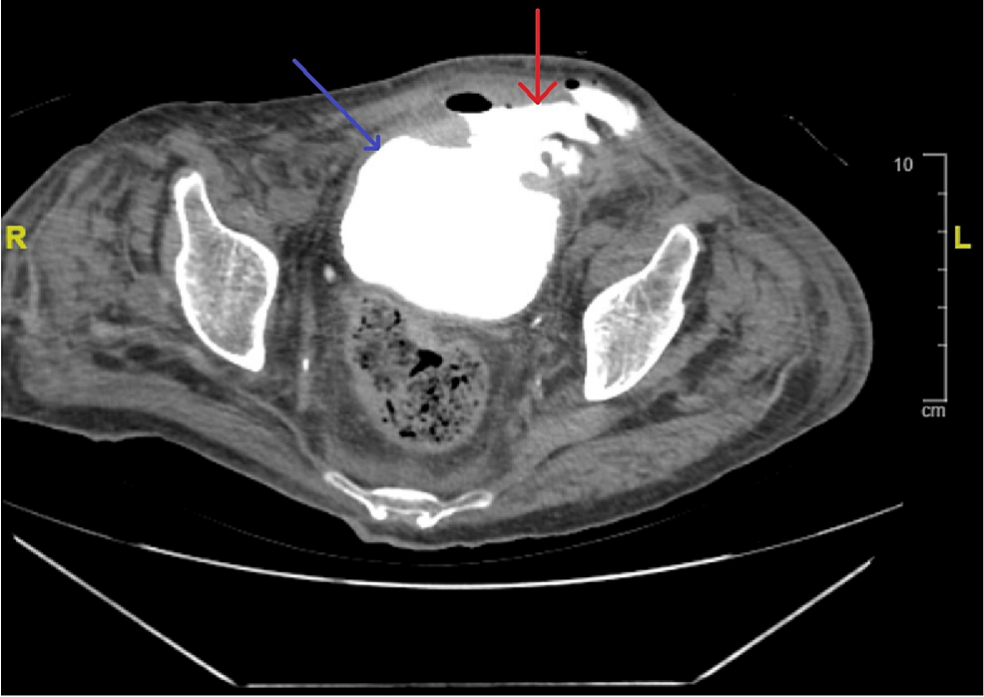
Computed tomography (axial view) shows a urinary bladder (blue arrow) anterior superior wall large defect, measuring about 4.3×4 cm, communicating with the anterior pelvic encysted fluid collection (red arrow).

A follow-up ultrasound the next day showed a 2 cm defect in the anterior wall of the urinary bladder (UB), minimal surrounding free pelvic collection, and both kidneys appearing normal in size, thickness, and echogenicity. No renal back pressure, cysts, calculi, or dilatation of the ureters were observed. A contrast-enhanced CT of the abdomen and pelvis revealed an anterior urinary bladder wall defect extending to the left lateral abdominal wall, abdominal wall abscess formation, and free pelvic fluid collection. Cystography with a pelvic CT scan confirmed communication between the anterior urinary bladder wall and left lateral abdominal wall, with complicated localized abdominal wall urinoma (Figures 1, 2).

Our patient suffered from the intraperitoneal bladder rupture. The patient underwent a surgical intervention. The general surgery team was involved in the beginning of the operation; they did the incision and drainage of the abdominal wall abscess, which revealed 20 cm × 8 cm of abdominal wall cavity extending to the bladder defect site. There were no other abdominal pathologies identified, such as fistulas. The wound was closed, dressed, and secured. Subsequently, the urology team performed a midline sub-umbilical incision, identifying and repairing a 3 x 4 cm defect in the anterior bladder wall. The procedure included the insertion of suprapubic and urethral catheters (16 Fr and 20 Fr, respectively), suture repair of the bladder wall, and verification of leak integrity. Closure procedures were performed in layers with appropriate suturing techniques. No histopathology sample was taken.

**Figure 3:**
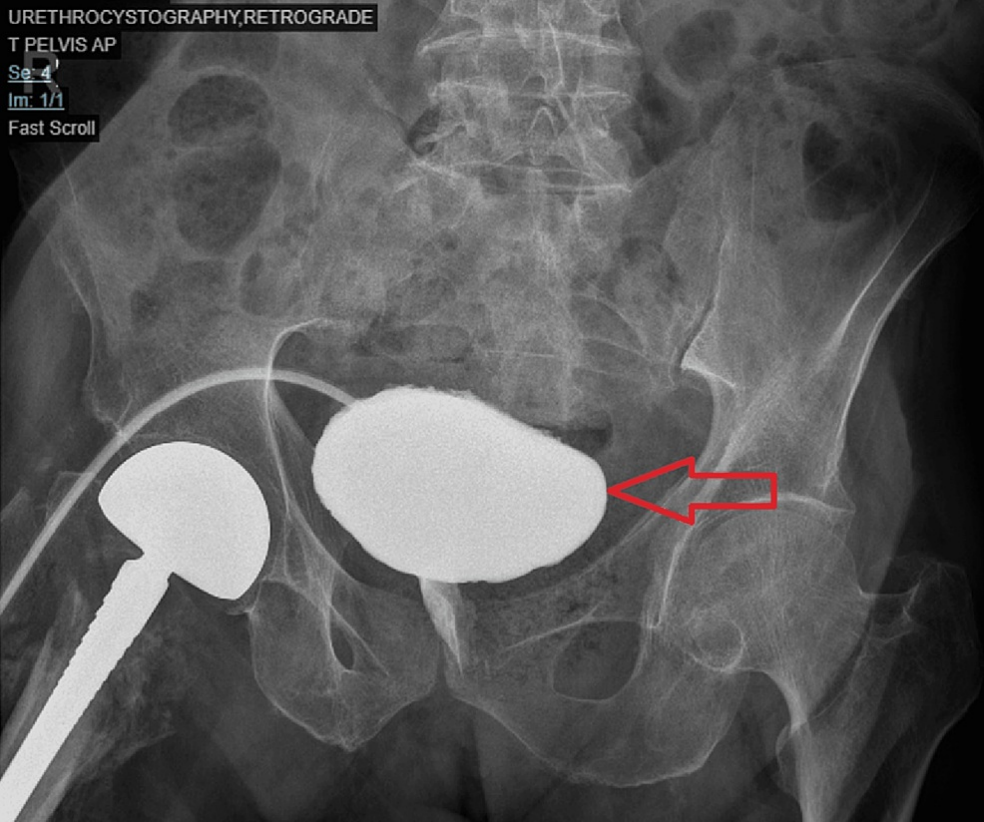
Retrograde urethrocystography with a red arrow showing the bladder three weeks post-surgery.

Post-operatively, the drain was removed after two days, with the last 24-hour drainage being less than 20 cc. The urethral catheter was removed on day five, and the patient was discharged on day six with a suprapubic catheter in place. Laboratory findings at discharge included Hgb: 7.74 g/dL, total leukocyte count (TLC): 7.96 x 10^3^/uL, PLT: 259k/uL, CRP: 61.3 mg/L, procalcitonin: 0.16 ng/mL, creatinine: 67.5 µmol/L, and lactate: 1 mmol/L (Table 1). Subsequent ascending cystography was performed after three weeks for a detailed investigation (Figure 3). A one-year follow-up revealed a single urinary tract infection treated with antibiotics; no further complications such as leakage, fistula, or any other issues were observed.

**Table 1:** Laboratory findings of the patient at discharge. N/A: not available.

Parameter	Emergency department presentation	Discharge	Normal range
Hemoglobin (g/dL)	10.2	7.74	13.8 to 17.2 (male)
White blood cells (10^3^/uL)	9.98	7.96	4.5 to 11.0
Platelets (k/uL)	218	259	150 to 450
Segmented neutrophils (%)	90	N/A	40-60%
C-reactive protein (mg/L)	270	61.3	<10 mg/L
Procalcitonin (ng/mL)	N/A	0.16	<0.5 ng/mL
Creatinine (µmol/L)	N/A	67.5	59-104 µmol/L (male)
Lactate (mmol/L)	N/A	1	0.5 to 2.2 mmol/L

## Discussion

SRUB is quite uncommon but a potentially fatal emergency. With less than 1% of bladder injuries, the incidence of SRUB is about 1 in 126,000. The weakening of the urinary bladder wall, predominantly caused by urine retention, is hypothesized to be responsible for the development of the SRUB. In individuals with SRUB, a number of predisposing factors have been seen, including but not limited to chronic urine retention, pregnancy, lower urinary tract obstruction, alcohol misuse, bladder dysfunction, bladder surgery, diverticulum, tumors, malignancy, infection, and inflammation. Poorly managed diabetes can cause urine retention, bladder dysfunction, and urinary tract infections, which are key risk factors for SRUB [6]. In accordance with Sisk and Wear's 1929 description, the condition exists if the bladder ruptures on its own accord without external stimuli and needs to be recorded as such [7]. In the literature, the age distribution for SRUB varies in different studies, but it is generally reported to occur in middle-aged to elderly individuals, with a median age ranging from 33 to 65 years old [8].

Abdominal pain or discomfort is a usual manifestation of SRUB, which has been recorded in numerous studies. Bladder rupture may occur more frequently in diabetics due to diminished bladder sensitivity, prolonged urine retention, and recurrent UTIs. Patients with diabetes are involved in many of the reported cases in the literature [9]. This is true in light of our case since our patient presented with severe abdominal pain and was a known case of diabetes as well. Similarly, Kabarriti et al. reported the case of a 36-year-old man who experienced sudden, severe stomach pain with no apparent cause [9]. At the time of his presentation, his blood sugar level was 1107 mg/dL. Initial abdominal imaging suggested that he had a significant pelvic growth that most likely originated in the bladder. He had an intraperitoneal perforation, which was revealed after further investigation, and consequently, he underwent an exploratory laparotomy and cystography [10]. Likewise, Eghbali et al. demonstrated the case of a 70-year-old diabetic woman with acute, diffuse abdominal pain and six-hour-long hematuria who presented to the emergency room [5]. Upon physical evaluation, generalized peritonitis was observed. Free air and fluid were visible in the abdominal cavity on multi-slice CT images of the abdomen and pelvis. The patient was taken to the operating room for an exploratory laparotomy after receiving the necessary resuscitation. At the bladder's dome, a 2 cm full-thickness bladder rupture was identified and treated [6].

Su et al. described that an abdominal CT scan can provide conclusive evidence of urinary bladder rupture [10]. However, CT might not be able to show a urinary bladder rupture without cystography or intravenous contrast following delayed imaging. It has been demonstrated that CT cystography, or contrast-enhanced CT with delayed imaging, is equally effective as traditional cystography for detecting bladder rupture. Additionally, straightforward procedures like comparing fluid concentrations of urea and creatinine with the corresponding serum values can help identify a case of urinary bladder rupture leading to ascites. A greater than 1.0 ratio of ascitic fluid creatinine to serum creatinine is strongly indicative of an intraperitoneal urine leak [11]. Furthermore, Lowe et al. agreed that urinary cystograms and abdominal CT scans can both provide diagnostic information. Although false-negative cystography with bladder perforation is not unusual, a cystogram is the preferred diagnostic test. When cystography is negative or ambiguous, the use of a CT scan to confirm the diagnosis is successful. An accurate, non-invasive approach for evaluating bladder pathology, particularly in patients with suspicion of bladder perforation, is the combination of CT and cystography. However, physician preferences and patient considerations all play a role in the overall approach decision [12]. Similarly, in our patient, all these diagnostic investigations, including CT with contrast and cystography with CT, were performed for the confirmatory diagnosis.

For treatment of SRUB, the vast majority of published case reports involve primary bladder repair, and for patients with intraperitoneal bladder rupture, management approaches involve performing an exploratory laparotomy, draining the urine from the peritoneum with a culture sample, washing the peritoneal cavity with a lot of physiological solution, stitching the ruptured bladder, and ensuring proper bladder and abdominal drainage. However, for some patients with favorable characteristics, conservative care might be taken into consideration. This covers the patient's clinical condition, symptoms, any minor complications, the lack of any obvious hematuria, and any infection symptoms. If conservative therapy fails, definitive surgical management should be made available [13]. In our case, surgical intervention was performed due to the complicated localized abdominal wall urinoma and the need for immediate management. Additionally, our patient had an intraperitoneal bladder rupture.

Intraperitoneal bladder rupture occurs when the bladder ruptures above the peritoneal reflection, which results in the leakage of urine into the peritoneal cavity. This can cause peritonitis, which is a serious infection. Intraperitoneal bladder rupture typically requires surgical repair. Extraperitoneal bladder rupture occurs when the bladder ruptures below the peritoneal reflection, which results in the leakage of urine into the extraperitoneal space, patients may feel a sudden sharp pain in the lower abdomen, difficulty or pain when urinating, blood in the urine, fever, nausea, and vomiting. This is a less serious condition, and it can often be managed conservatively with a urinary catheter. The prognosis for both types of bladder rupture is generally good, but it depends on the severity of the rupture and the promptness of treatment [14]. Our case was efficiently managed with the repair of the urinary bladder. For follow-up in this case, it was decided to conduct an ascending cystography in order to conduct a more thorough investigation and find any potential leaks. The patient had an episode of a UTI and was successfully treated with IV antibiotics. His suprapubic catheter was functioning well with no problems. Counseling the patient on future risks is difficult in the absence of any etiology. However, it was advised to seek emergency medical care if experiencing the typical symptoms of SRUB, such as sudden-onset abdominal distension, discomfort, and decreased urine production.

## Conclusions

SRUB is an exceedingly rare clinical condition that poses a life-threatening challenge; therefore, early diagnosis and prompt management are crucial in addressing this issue. Significant attention should be given when diabetic patients present with sudden severe abdominal pain and urinary retention. Furthermore, to avert fatal complications, any necessary laparoscopy or laparotomy procedures must not face delays and should be executed immediately.
